# Compliance with Barrier Gestures during COVID-19 Pandemic as a Function of the Context: A Longitudinal Observational Survey at the University of Liège

**DOI:** 10.3390/ijerph191811523

**Published:** 2022-09-13

**Authors:** Gianni Parisi, Véronique Renault, Marie-France Humblet, Nicolas Ochelen, Anh Nguyet Diep, Michèle Guillaume, Anne-Françoise Donneau, Fabrice Bureau, Laurent Gillet, Anne-Catherine Lange, Fabienne Michel, Sébastien Fontaine, Claude Saegerman

**Affiliations:** 1 Research Unit in Epidemiology and Risk Analysis Applied to Veterinary Sciences (UREAR-ULiège), Fundamental and Applied Research for Animal and Health (FARAH) Center, University of Liège, 4000 Liège, Belgium; 2 Vétérinaires et Agronomes Sans Frontières, 69007 Lyon, France; 3 Unit of Biosafety, Biosecurity Unit and Environmental Licenses, Department of Occupational Safety and Hygiene, University of Liege, 4000 Liege, Belgium; 4 Faculty of Veterinary Medicine, Liège University, 4000 Liège, Belgium; 5 Biostatistics Unit, Liège University, 4000 Liège, Belgium; 6 Department of Public Health, Faculty of Medicine, Liège University, 4000 Liège, Belgium; 7 Risk Assessment Group COVID-19, Liège University, 4000 Liège, Belgium; 8 COVID-19 Platform, Liège University, 4000 Liège, Belgium; 9 Laboratory of Cellular and Molecular Immunology, GIGA Institute, Liège University, 4000 Liège, Belgium; 10 Fundamental and Applied Research for Animal and Health (FARAH) Center, Liège University, 4000 Liège, Belgium; 11 Laboratory of Immunology-Vaccinology, Liège University, 4000 Liège, Belgium; 12 Récolte et Analyse des Données et Information d’Utilité Stratégique (RADIUS), Liège University, 4000 Liège, Belgium; 13 Institute for Research in Social Sciences (IRSS), Faculty of Social Sciences, University of Liège, Place des Orateurs 3, 4000 Liège, Belgium

**Keywords:** adherence, barrier gesture, COVID-19, compliance, SARS-CoV-2

## Abstract

During the COVID-19 pandemic, barrier gestures such as mask wearing, physical distancing, greetings without contact, one-way circulation flow, and hand sanitization were major strategies to prevent the spread of SARS-CoV-2, but they were only useful if consistently applied. This survey was a follow-up of the first survey performed in 2020 at the University of Liège. We aim to evaluate the compliance with these gestures on campuses and examine differences in the extent of the compliance observed in different educational activities and contexts. During 3.5 months, the counting of compliant and non-compliant behaviors was performed each week in randomly selected rooms. Using data collected during both surveys (2020 and 2021), binomial negative regression models of compliance depending on periods (teaching periods and exam sessions), type of rooms, and campuses were conducted to evaluate prevalence ratios of compliance. The percentage of compliance in this second survey was the highest for mask wearing and physical distancing during educational activities (90% and 88%, respectively) and lowest for physical distancing outside educational activities and hand sanitization (45% and 52%, respectively). Multivariate analyses revealed that the compliance with most gestures was significantly higher in teaching rooms than in hallways and restaurants and during exam sessions. The compliance with physical distancing was significantly higher (from 66%) in auditoriums, where students had to remain seated, than during practical works that allowed or required free movement. Therefore, the compliance with barrier gestures was associated with contextual settings, which should be considered when communicating and managing barrier gestures. Further studies should specify and confirm the determining contextual characteristics regarding the compliance with barrier gestures in times of pandemic.

## 1. Introduction

In December 2019, the first case of coronavirus disease 2019 (COVID-19) was reported in the province of Wuhan, China [1]. Since then, severe acute respiratory syndrome coronavirus type two (SARS-CoV-2), responsible for the disease, has spread all over the world causing one of the major pandemics of the century. Despite a significant proportion of infections by SARS-CoV-2 being asymptomatic [2] or causing moderate effects, COVID-19 can, especially in people with comorbidities, present severe forms, characterized by breathing difficulties requiring treatment in an intensive care unit, and may lead to death. This, associated with its high contagiousness, has resulted in more than 6.425 million mortalities (12 August 2022) [3].

At the beginning of the COVID-19 pandemic, no vaccine was available, and the development of such vaccines required different phases of clinical trials. The only possibility to reduce the transmission of the virus and the overcrowding of intensive care units was to implement mitigation measures, essentially based on the adoption of barrier gestures by the population.

By the end of 2020, vaccines became available [4] for people at higher risk of developing complications and healthcare workers. Then, during the year 2021, they became slowly available for the general population. By that time, vaccines had demonstrated their ability to reduce the number of cases of COVID-19 [5,6] but little was known about their ability to reduce the virus transmission once a person was infected. Given this uncertainty and the time required to reach a broad vaccination coverage, recommendations for the use of barrier gestures were maintained in Belgium.

The efficacy of barrier gestures, whether they are mandatory or not, depends on the adherence and their effective implementation by the population. In addition to personal beliefs, perceptions of the usefulness of each measure in preventing the virus transmission, and the effort needed to adhere to the gesture, social influence and the facilitating conditions play a determining role [7,8]. The former refers to the extent to which individuals perceived that it was the norm to adopt the barrier gestures, while the latter refers to the availability of the organizational and technical infrastructure that supports the implementation [8]. Although several studies focused on the acceptability, the adhesion, and the self-declared compliance of the population to barrier gestures [9,10,11,12,13,14,15], there are fewer studies assessing the compliance with barrier gestures directly by observational methods [16,17,18,19] and/or taking into account the contextual factors as mentioned. This has limited our understanding of how the organization and management of barrier gestures can be efficiently improved, from a public health and institutional perspective.

At the beginning of the 2020–2021 academic year (from week 39 to week 43 of 2020), at the University of Liège (Belgium), while a return to on-site educational activities was announced after one academic year of remote teaching and learning due to the lockdown, a survey took place to investigate the extent to which barrier gestures were applied [20]. As described previously [20], Belgian authorities defined a color code to organize teaching modalities in universities during this pandemic period, according to the epidemiological situation (green, yellow, orange, and red) (Table A1). The first survey investigated the compliance rate with mask wearing, circulation flow, hand sanitization, physical distancing, and greetings without contact by structured observations in the auditoriums, hallways, and restaurants of the university. It lasted only five weeks, four while Belgium was in the yellow code one while it was in the orange code. The survey ended when the red code had come into force, and teaching had been replaced by distance learning. This first study had underlined that an improvement was deemed necessary in terms of compliance with hand sanitization, circulation flow, and physical distancing outside teaching activities.

During this first survey, the orange code only lasted one week, and no further observations were conducted because the red code was being applied in force. However, it is of significance to assess to what extent barrier gestures are applied when the epidemiological situation is considered critical, as behaviors can differ from one situation to another. In addition, as the epidemiological situation becomes worst (i.e., switching to the orange code and, even more so, to the red code), the teaching activities preferably kept as face-to-face, namely practical works, could hardly (if at all) be carried out remotely. These practical works have not been the subject of any particular observation, although compliance behaviors may differ in this context from auditorium teaching activities. Finally, no observation could be performed in the first survey during the exam sessions that, nevertheless, represented one-third of the academic calendar for the campus. Henceforth, the aim of the present study is to monitor the compliance of the university population with barrier gestures in the different contexts mentioned above, in addition to the previous study, in order to obtain a broader picture of the compliance of the study population during an academic year.

The second aim of the present study was to determine the contextual factors associated with compliance/non-compliance behaviors, which included the color codes presenting the risk levels applied to each teaching period by the regional authorities, the types of educational activities, the types of rooms and campuses, and the timing of observation. This second survey started in March 2021, when a return to a 20% face-to-face teaching was announced.

## 2. Materials and Methods

### 2.1. Study Population

The population studied was the university community members present on site during the study period. As homeworking was recommended/mandatory when possible, and a part of teaching activities went online, it was complicated to accurately characterize this population on-site. Information presented here was drawn from institutional data on the university community in general. The university population consisted of mainly students (almost 27,000 students, i.e., 83% of the population), whereas employees, researchers, and academic staff represented 17% of the population. Among these students, 1% were attached to the campus of Arlon, 6% to the campus of Gembloux, 27% to the campus of Liège Center, and 67% to the campus of Liège Sart-Tilman.

### 2.2. Survey

The compliance with five barrier gestures—namely greetings without contact, physical distancing, mask wearing, following a circulation flow, and hand sanitization—was determined by structured observation, following a methodology similar to the one used during the previous study (2020) [20]. The 30 observers, who were students, followed an online training, received an observation guideline, and had to pass an observation test (with at least 75% correct answers) prior to the beginning of their field observation in the study. This was to ensure that barrier gesture compliance was interpreted in a similar manner by all observers. The tasks were also randomly attributed to the observers on a weekly basis. They then had to collect the data using a structured form and following the observation guideline.

Each week, at each campus of the University of Liège (*n* = 4, i.e., Arlon, Gembloux, Liège Center, and Liège Sart-Tilman), rooms were randomly drawn from the lists of the rooms where educational activities were planned. An observation session of the application of barrier gestures (i.e., a period during which one observer successively observed the five barrier gestures in a specific room) was carried out in each room when occupied. Different types of rooms were distinguished: (i) auditoriums (where lectures were given and students were seated during the activity and had few interactions with one another, with the professor or any educational material), (ii) rooms where practical works were performed (where students might have to move, interact with each other, with the professor or any educational material), and (iii) restaurants and cafeterias. Clinical teaching activities were excluded from observations, as they could involve people from outside the university. Due to the orange and red codes, many educational activities switched to an online mode. Classroom occupancy and class hour modifications were not systematically reported. As a consequence, when an observer encountered an empty room, they had to carry out the observation in the next closest occupied classroom. If no classroom was occupied nearby, the observation was alternatively performed in the building hallway.

For each gesture, the observer counted the number of people who complied with the expectation and the number of people who did not fully or partly comply with the observed gesture, as described in the next section.

- Physical distancing

In hallways and restaurants, the observer counted, for 10 min, the number of people that passed by (or waiting in line in the restaurant) and kept a distance of 1.5 m between each other and the number of people that did not. During practical works, as the number of participants was rather small and fixed, the count was done for all students in the room and repeated three times to obtain a mean number during this session. The 1.5 m distance was estimated by the observer, using floor markings when available.

In auditoriums, the observer counted the number of students that left an empty chair on both sides and the number of students that did not.

- Mask wearing

In hallways and restaurants, the observer counted, for 10 min, the number of people that passed by (or waiting in line) and wore a mask correctly and the number of people that did not. During practical works, as for physical distancing, the count was done three times for all students present in the room. In auditoriums, the count was done on a fixed number of seated students but not necessarily for all in the auditorium, as it could be difficult to assess if a mask was correctly worn by students who were seated farther away from the observer. The mask wearing gesture was considered compliant when the mask was worn and effectively covered the nose and the chin.

- Greetings without contact

At the entrance to the observed place, the observer counted, for 10 min, the number of greetings without contact, i.e., completely without contact or using the elbow, and the number of greetings with contact.

- Hand sanitization

The observer positioned themselves in front of a hydroalcoholic gel dispenser and, for 10 min, counted the number of people that used the gel dispenser and the number of people that did not, upon entering or leaving the room or the building. For practical works, students were observed upon entering and exiting the room. Moreover, when soap and sink were available, handwashing was included as a compliance, when it was possible to observe.

- Circulation flow

For each 10 min observation period of entries or exits of the building or the room, the observer counted the number of people that followed the direction as indicated and the number of people that did not. Circulation flow was not observed during practical works.

Each time an observer had to use a chronometer to count the people moving; if there were too many people, so it was too difficult to proceed with simultaneous counting of compliant and non-compliant gestures, the observation was split into two five-minute countings: one to count compliances and the other to count non-compliances.

The results of all observations were encoded online via Lime Survey, and a weekly review of these data (i.e., a data verification to detect possible encoding problems or inconsistencies) was performed by the first author. This survey took place from week 11 to week 25 of 2021, with an interruption during weeks 14 and 15 because of spring break. All sessions were performed when the orange code was in force at the University, except weeks 13 to 15, when the red code was in force. The exam session took place from week 21 to week 25. During this period, the observations focused on the auditoriums where written exams were organized. No observation was planned for practical and oral exams, as it concerned a limited number of students and could not be carried out without disturbing the observed people. During this period, an authorization to observe was requested and approved from the professors in charge of the exam.

### 2.3. Statistical Analysis

The statistical analyses were conducted with R software, version 4.1.2 (R Core Team, Vienna, Austria, 2021) [21].

Firstly, a descriptive analysis of the observation sessions and observed compliance behaviors was carried out for the data collected during this second survey (2021). Based on a method developed, presented, and used previously [20], an overall weighted score (OWS) was calculated for the compliance with barrier gestures, for the data collected during 2021. Briefly, 38 international professionals with expertise on COVID-19 were asked to weight barrier gestures according to their importance, by distributing 100 points. The most important gesture, therefore, received more points. The OWS was defined as the sum of the products of the median weights of each gesture, and the level of compliance and ranged from 0 to 100.

Secondly, for each barrier gesture, a multivariate binomial negative regression on the binarized gesture (compliant vs. non-compliant) depending on the type of rooms, campus, and period of observation. For the circulation flow and hand sanitization, the timing of observation (entry vs. exit) was added to the regression. As no multivariate analysis had been performed on the data from the first survey (2020), we incorporated the previously collected data into the present study and performed the analysis at the multivariate level. The approach allowed us to identify the contextual factors associated with enhanced or decreased compliance, taking into account the interactions between the variables (e.g., increased number of observation sessions in corridors during weeks when many activities initially planned in face-to-face were cancelled, which were probably those when the epidemiological situation was at its worst). Six observation periods were considered for the regressions. For the first survey (2020) [20], the yellow code teaching period (from week 39 to week 42 of 2020) and the first orange code teaching period (week 43 of 2020) were considered. For the second survey (2021), the following periods were considered: (i) the second orange code teaching period (from week 11 to week 12 of 2021), (ii) the red code teaching period (during week 13 of 2021), (iii) the third orange code teaching period (from week 16 to week 20 of 2021), and (iv) the exam session (from week 21 to week 25). The exam session was organized during the orange code was activated but was considered as a different period, given the different types of on-site activities.

Variables included in the multivariate models were first selected based on the *p*-value of the univariate model (*p*-value < 0.2) [22]. A backward stepwise selection method based on the Akaike Information Criterion was then performed. The prevalence ratios of compliances with gestures were calculated for all variables included in the multivariate models. The prevalence ratio of compliance was defined as the ratio between the proportion of compliant behaviors when the studied variable took a modality (e.g., room observed = practical work room) and the proportion of compliant behaviors when the variable treated was the reference modality, to which it was compared (e.g., observed room = auditorium).

## 3. Results

### 3.1. Observation Sessions of 2021

A total of 314 observation sessions were performed during 2021 (Table 1), in which more than 28,000 behaviors were observed (Table 2). As many of the teaching activities were organized in online mode, many rooms were found empty by the observers; consequently, the planned observation sessions took place in hallways (40%). When the red code was implemented (week 13), restaurants were closed completely; hence, few observations session were realized in this setting (1%).

A functional hydroalcoholic gel dispenser was present at the entry and the exit of the building/room in 93% and 74% of observation sessions, respectively. A circulation flow was observable in 76% and 73% of auditoriums and hallways, respectively. During observation sessions performed in auditoriums and during practical works, windows and doors stayed were opened at least half of the time in 36% and 51% of observation sessions, respectively. The movement of the students in the room was necessary in 48% of the practical works (i.e., the students could not remain seated during these practical works).

### 3.2. Compliance with Barrier Gestures and Overall Weighted Score of Compliance for the Survey of 2021

For the whole period in 2021 (from week 11 to week 25), the compliance rate was 68% for greetings without contact, 52% for hand sanitization, 72% for circulation flow, 90% for mask wearing, 88% for physical distancing during teaching activities, and 45% for physical distancing outside teaching activities (Table 2). In 49% of all observation sessions, all the persons observed were compliant regarding mask wearing. Regarding non-compliance, observers estimated that the main non-compliance behaviors were that the mask did not cover the nose in 74% of sessions, there was absence of a mask in 24% of sessions, and the mask did not cover the chin in 2% of sessions.

When comparing compliance rates with the first survey (see Table A2), no difference was observed for compliance with physical distancing, neither during nor outside of teaching activities. Though, the compliance with greetings without contact was 15% lower during the second survey, while the compliance with hand sanitization, circulation flow, and particularly mask wearing increased by 8%, 7%, and 11%, respectively.

The OWS of compliance with barrier gestures for the study period reached 68.2.

### 3.3. Compliance Depending on Observation Periods, Room Types, and Campuses

The prevalence ratios calculated using multivariate analysis are shown in Table 3. Compliance with hand sanitization was significantly lower (from 50% to 56%) when exiting a building or a classroom than during the entry, as well as compliance with circulation flow (from 4% to 13%).

Except for hand sanitization, compliance with all gestures was significantly lower in hallways compared to auditoriums, reaching a 48% to 53% decrease for physical distancing. Similarly, in restaurants and cafeterias, except for greetings without contact, compliance with all gestures was significantly lower, reaching a 49% to 56% decrease for physical distancing. Still, compared to auditoriums, observations during practical works showed a significantly higher compliance with hand sanitization (from 26% to 56%) but a significantly lower compliance with physical distancing (45% to 34%).

Compared to the Liège Sart-Tilman campus, the Gembloux and Arlon campuses presented a significantly lower compliance with circulation flow (from 9% to 17% and from 28% to 55%, respectively). The compliance with hand sanitization on these two campuses was significantly higher (from 7% to 36% for Arlon and from 19% to 42% for Gembloux) compared to the Liège Sart-Tilman campus, while it was slightly and significantly lower in Liège Center (from 2% to 12%). Regarding the compliance with mask wearing, the only significant difference between campuses was a decrease from 5% to 21%, for Arlon compared to Liège Sart-Tilman.

When considering the different periods, a significantly higher compliance with mask wearing was noticed for all periods, except during the red code period as compared to the yellow code period. For the red code period, the only significant difference appeared for physical distancing, which was 3% to 37% lower compared to the yellow code period. Compliance with physical distancing also significantly decreased for the first orange code period (between 4% to 18%) but significantly increased during the second orange code period and the exam session (between 9% to 35% and between 5% to 14%, respectively). The compliance with hand sanitization was significantly higher during the first orange code period and the exam session compared to the yellow code period (between 9% to 30% and between 23% to 40%, respectively). During the third orange code period and the exam session, i.e., the last two observation periods, greetings without contact were significantly less complied with compared to the yellow code period, making it the only less complied gesture during the exam session, as the compliance with circulation flow was significantly higher (from 10% to 24%) during this period than during the yellow code period.

## 4. Discussion

### 4.1. Compliance with Barrier Gestures and Overall Weighted Score

If one compared the raw compliance rates between this survey and the first one [20], it seemed that an effort was made in terms of compliance with the use of hydroalcoholic gel, circulation flow (which was encouraged at the end of the first survey), and, even more so, with mask wearing, while a decreased compliance was observed for contactless greetings. The more frequent non-compliance with the use of mask wearing was misplacing the masks on the face, which was also shown in other an observational study [23]. The compliance OWS was shared each week with the university risk assessment group, to provide a global overview of ‘on-site’ compliance with barrier gestures and the possible need to adapt communication. Compliance OWS was globally the same for both surveys, despite some differences observed for compliances with each gestures individually. The decreased compliance with contactless greetings, which was classified as the second-most-important barrier gesture by COVID-19 experts, was offset by an improved compliance with hand sanitization, circulation flow, and mask wearing. Nevertheless, as the second survey covered quite different observation periods (orange/red codes, exam session, etc.), it was worth examining the compliance further, with the help of the models produced.

### 4.2. Prevalence of Compliance According to Periods, Room Types, Campuses, and Timing of Observation

The decreased compliance with circulation flow and hand sanitization highlighted during exiting from rooms was consistent with the previous univariate analysis [20] and could be attributed to the fact that the communication about barrier gestures focused mainly on their implementation upon entering buildings or rooms, which was observed not only in the context of the university but also in other situations of daily life (e.g., a hydroalcoholic gel dispenser at the entry of stores). Posting a sign related to hydroalcoholic gel dispensers at the exit could increase awareness and reinforce the use of the dispensers when exiting.

In hallways and restaurants, the compliance with gestures appeared to be lower in both contexts, except for contactless greetings in restaurants and hand sanitization in hallways. This could be due to the fact that, in a teaching context, the professors could ensure a certain control on barrier gestures’ implementation by the students, which was not the case in a less formal context. The decrease in compliance was particularly high for physical distancing; it was about two-fold lower in hallways and restaurants compared to auditoriums. Such a decrease could result, apart from the potential control of barrier gestures implementation by academics during teaching activities, from the difficulty to constantly evaluate and keep a distance with each other in such a situation. Indeed, when attending an ex cathedra course, sitting and leaving an empty chair on each side was rather easy, if the number of participants was not too high in relation to the room capacity (which was taken into account when booking the classrooms). In contrast, keeping a 1.5 m distance from other people, while standing in a room, could be challenging, especially if there was no exact distance marking or indication in the surroundings, if the people were moving, and/or if there was a large number of people in a small space. Besides, keeping a distance required other individuals’ cooperation [24], and distinct individual perceptions could lead to coordination difficulties. In an observational study conducted on students seated in a university library in Canada at the same period of our second survey (March–April 2021), a relatively high compliance with both physical distancing and mask wearing (78%) was also documented [25].

The difficulty of keeping a distance when moving could also explain the lower compliance with this gesture during practical works, compared to in auditoriums, as students moved freely in about half of the practical works. The higher compliance of hand sanitization when entering or exiting practical works than when entering or exiting auditoriums could be a good indication, since, during practical works, the use of shared materials was more likely to foster hand-borne infection. This higher compliance could be explained by a higher perceived risk of contamination when using shared materials, as well as the pre-established hygiene habits specific to the teaching activities in question (e.g., mandatory handwashing when handling biological materials).

The context, i.e., the type of rooms observed, seemed to be of importance in terms of compliance with barrier gestures. Therefore, it could be interesting for the authorities to communicate or emphasize the importance of continuing to comply with gestures in different contexts. Nevertheless, in some given situations, certain gestures could not be easily complied with, e.g., physical distancing during practical works. Therefore, stressing the application of other gestures, such as mask wearing, could be a compensating solution.

When looking at the compliance with circulation flow at the campus level, it was lower for both the Arlon and Gembloux campuses compared to the Liège Sart-Tilman campus, while compliance with hand sanitization was higher for the former. Such higher compliance with hand sanitization could be due to differences in the perception of the importance of the gestures in the population of these campuses, as they were both welcoming students whose courses were related to life sciences (i.e., bioengineering and environmental sciences), but could also be only apparent and not result from an actually lower use of hydroalcoholic gel. Indeed, it was reported that in some university locations, hydroalcoholic gel dispensers were placed in a series (e.g., at the entrance to a building and at the entrance to different rooms or at the exit to rooms and at the exit to a building). In such cases, people passing by two consecutive dispensers did not necessarily use both, which was understandable and should not be considered as a non-compliant behavior. The gesture observation at these dispensers could lead to an underestimation of the actual compliance, given the fact that some people not using the gel at the observed place had just done it a moment earlier. In smaller campuses (Arlon and Gembloux), the number of hydroalcoholic gel dispensers arranged in a series could be lower, which could increase the apparent compliance, bringing it closer to the actual compliance.

The higher compliance with mask wearing for all periods, except the red code teaching period compared to the yellow code teaching period, seemed to indicate a better integration of the practice in daily life compared to the beginning of the academic year. Such an increase was also positive, given the low compliance with physical distancing outside teaching activities. Such an improvement of observed compliance with mask wearing between the second half of 2020 and the first half of 2021 was also evidenced in public spaces in a Spanish study [23]. The absence of significant differences between the yellow code teaching period and the red code teaching period was possibly due to the fewer observation sessions achieved during the latter.

It might seem surprising that, during this red code teaching period, while the authorities directed their attention to a more critical epidemiological situation, the compliance with physical distancing was lower compared to the yellow code teaching period and the second orange code teaching period. This could be explained by the transition of all activities previously on-site to remote learning, except some practical works on-site with a nature that might make compliance with physical distancing more difficult. Besides, 60% of practical works during this period required the free movement of students.

When looking at the exam session compared to the yellow code teaching period, the compliance was higher for all gestures, except contactless greetings. During the exam session, a larger part of the university population returned on-site for organizational reasons. This prompted the university authorities to implement special measures in some places to ensure compliance with barrier gestures, e.g., distribution of surgical-type masks at the entrance of buildings and presence of staff at the entrance of buildings, to ensure that the people entering used hydroalcoholic gel and wore a surgical-type mask. In addition to these measures, professors could have played a greater role in controlling compliance with barrier gestures, due to the nature of exams and a more massive return to the campuses. It is also important to note that during exam sessions in a non-pandemic context, students are usually seated apart to prevent cheating. Such a habit has, therefore, favored the application of physical distancing during that period. Interestingly, during the exam session, the only barrier gesture with a lower compliance compared to the yellow code teaching period was a gesture that was difficult to control, i.e., contactless greetings. Already decreasing during the third orange code teaching period, the lower compliance with contactless greetings could reflect a lesser attention paid by students to comply with barrier gestures beyond any controlled by dedicated persons. Such a decline of compliance with mask wearing over time was reported previously [19]. In the present situation, the acceleration of vaccination deployment in the population (19.3% of Walloon adults were fully vaccinated by 24 May 2021 vs. 40.6% by of 21 June 2021 [26]) and the decrease in the number of new cases each week (from 4585 in week 21 to 615 in week 25 vs. 9742 in week 11 in Wallonia [27]) may have induced a lower risk perception. As highlighted in previous studies [10,11,19], a lower risk perception was associated with a lower compliance with barrier gestures and could, therefore, explain a relaxation of their implementation. That said, this hypothesis should be confirmed, as a study realized in 10 universities around the world [28] showed that compliance with barrier gestures was not uniformly influenced by the same factors, given the underlying differences between hygiene measures and measures related to physical distancing. This decrease in compliance with greetings without contact during the two last periods could, therefore, be independent and not reflect a general decrease in compliance with other gestures. Vaccines have shown a certain capacity to reduce the transmission of SARS-CoV-2 variants, i.e., the Alpha variant and, to a lesser extent, the Delta variant, but do not completely prevent the spread [29]. Such a capacity to reduce virus transmission decreases a few months after vaccination [29]. Moreover, the Omicron variant, which is currently predominant [30], seems to be able to escape antibodies elicited by vaccination [31,32]. Since the response to the vaccine depends on the variant, and given that gestures such as physical distancing, mask wearing, and handwashing have shown a certain capacity to reduce transmission [33,34,35], barrier gestures do not seem obsolete yet and can be, along with proper indoor ventilation [36], a good non-variant-specific support for vaccination, to reduce virus circulation. As non-specific means of control, they have a positive impact on the circulation of other viruses such as influenza [37,38], so can be used during future pandemics.

This study had some strengths. One of them was the involvement of students employed in the data collection process, as their presence on-site as community members was relatively well-accepted by the observed population. This would not necessarily have been the case with external observers, who could have been perceived as a supervisory authority and whose presence, in addition to arousing a certain mistrust, could have distorted the behavior observed. To the authors’ knowledge, ours is the first study to longitudinally monitor compliance with these five barrier gestures by direct observations and over such a long period. The use of direct structured observation avoids the biases inherent in carrying out a survey by questionnaires, such as the framing effect and social desirability [39]. Finally, the study allowed for observations in different situational contexts within the university itself, i.e., teaching activities vs. hallways and the teaching period vs. the exam period.

The study has also some limitations, notwithstanding. First, even if rooms were selected randomly, it was difficult to ensure the perfect representativeness of observations each week, as it was not possible to determine to what extent the activities were concentrated in the different types of rooms; indeed, the number of students present in each room was variable. The fact that observations of hallways replaced observations of empty rooms made the control of the types of rooms even more difficult. That said, the multivariate model allowed for adjustment given the types of rooms observed. Secondly, the presence of the observer could have led to an enhanced compliance with barrier gestures. Indeed, being aware of the presence of people observing their behaviors, professors could have encouraged students to apply the barrier gestures. However, when a professor explicitly referred to the presence of the observer to foster compliance, in order to only consider authentic and unprompted behaviors, the observation was cancelled. Thirdly, since the study was conducted in a relatively controlled environment and within a population with a rather high level of education, it probably achieved a higher health literacy than that of the general population. This implies that the observed compliance may have been higher than what would have been observed in the general population and in other less controlled contexts. The findings could, therefore, be generalized for similar populations, and the methodology can be transposed to other contexts (e.g., markets, shops, meetings, or public transportations), which could make it possible to confirm the findings in different populations.

Finally, as mentioned before, the fact that hydroalcoholic gel dispensers were located close to each other had a certain impact on the apparently decreased compliance with hand sanitization.

## 5. Conclusions

The types of rooms and activities seemed to influence the compliance with barrier gestures. Compliance was generally higher during teaching activities and exams, where the presence of an authority could have been decisive, whereas a lesser extent of compliance in public spaces as hallways and restaurants was observed. Compliance with physical distancing seemed to be higher during lectures where students remained seated and to be lower when free movement was possible or unavoidable (in hallways, restaurant queues, and practical works). That said, one can understand that the compliance with barrier gestures depends not only on individuals’ characteristics and beliefs but also on the context of observation. In other words, the social influence and the availability of technical infrastructure can be significant determinants. To put it differently, a high compliance was more likely once individuals had an opportunity to adhere to the barrier gestures with no significant effort required or with fewer difficulties entailed, especially given the influence of the significant others around them, e.g., the professor in charge.

In order to improve the compliance of individuals with the measures implemented to control the spread of pathogens, it is recommended to adapt the communication and guidelines to the context. Suggestions include focusing communication on complementary measures when one gesture could not easily be adhered to, insisting on continuing to comply with barrier gestures, or raising awareness among individuals to remind them of the important rules in situations deemed critical, as was done by the stewards during the exam session. Further studies are strongly recommended to clarify and confirm the determining characteristics of the different contexts in the application of barrier gestures. In so doing, our knowledge of how to effectively manage barrier gestures could be further enhanced.

## Figures and Tables

**Table 1:** Observation sessions of barrier gestures during both surveys (2020 and 2021).

Variable	Survey of 2020 (*n* = 525)		Survey of 2021 (*n* = 314)		Total (*n* = 839)	
	*n*	%	*n*	%	*n*	%
Campus						
*Arlon*	39	7.4	13	4.1	52	6.2
*Gembloux*	35	6.7	19	6.1	54	6.4
*Liège Sart-Tilman*	268	51.1	181	57.6	449	53.5
*Liège Center*	183	34.9	101	32.2	284	33.8
Type of room						
*Auditorium*	362	69	134	42.7	496	59.1
*Hallways*	106	20.2	126	40.1	232	27.7
*Restaurant*	57	10.9	4	1.3	61	7.3
*Practical work*	0	0	50	15.9	50	6
Period						
*Yellow code teaching period*	432	82.3	/	/	432	51.5
*First orange code teaching period*	93	17.7	/	/	93	11.1
*Second orange code teaching period*	/	/	39	12.4	39	4.6
*Red code teaching period*	/	/	13	4.1	13	1.5
*Third orange code teaching period*	/	/	185	58.9	185	22.1
*Exam session*	/	/	77	24.5	77	9.2

**Table 2:** Observed compliance rate of barrier gestures from week 11 to week 25, 2021.

Barrier Gesture	Number of Compliant Observations	Total Number of Observations	Compliance Rate (%)	95% CI (%)
Contactless greetings	1419	2097	67.7	65.7–69.7
Hand sanitization	3448	6689	51.5	50.3–52.7
Circulation flow	3072	4249	72.2	70.9–73.6
Mask wearing	7194	7991	90.0	89.4–90.7
Distancing in auditoriums and during practical works	4700	5355	87.8	86.9–88.6
Distancing in hallways and restaurants	980	2161	45.3	43.3–47.4
Distancing (total)	5680	7516	75.6	74.6–76.5

*n* = number, CI = confidence interval.

**Table 3:** Binary binomial negative regression prevalence ratios of the compliance with barrier gestures during both surveys (2020 and 2021) (95% CI).

	Circulation Flow	Contactless Greetings	Hand Sanitization	Physical Distancing	Mask Wearing
**Period (reference: yellow code teaching period)**					
First orange code teaching period	0.964 (0.870–1.065)	1.104 (0.973–1.249)	1.195 (1.095–1.302) *	0.890 (0.823–0.962) *	1.079 (1.009–1.154) *
Second orange code teaching period	1.050 (0.923–1.19)	1.033 (0.855–1.237)	1.089 (0.957–1.234)	1.213 (1.090–1.347) *	1.172 (1.072–1.280) *
Red code teaching period	0.947 (0.519–1.566)	1.063 (0.774–1.430)	0.933 (0.748–1.153)	0.784 (0.627–0.970) *	1.045 (0.899–1.209)
Third orange code teaching period	1.044 (0.965–1.128)	0.857 (0.771–0.951) *	0.964 (0.889–1.045)	1.021 (0.961–1.083)	1.084 (1.027–1.143) *
Exam session	1.169 (1.102–1.239) *	0.786 (0.711–0.867) *	1.314 (1.233–1.399) *	1.091 (1.046–1.138) *	1.158 (1.109–1.209) *
**Campus (reference: Liège Sart-Tilman)**					
Arlon	0.579 (0.455–0.724) *	1.049 (0.891–1.228)	1.207 (1.069–1.357) *	/	0.868 (0.793–0.948) *
Liège Center	0.952 (0.905–1.002)	0.953 (0.885–1.024)	0.925 (0.877–0.977) *	/	1.026 (0.991–1.062)
Gembloux	0.828 (0.752–0.910) *	1.120 (0.995–1.256)	1.300 (1.189–1.418) *	/	1.058 (0.998–1.122)
**Type of room (reference: auditoriums)**					
Hallways	0.809 (0.759–0.863) *	0.888 (0.808–0.975) *	0.979 (0.916–1.046)	0.495 (0.469–0.522) *	0.886 (0.845–0.928) *
Restaurants	0.924 (0.857–0.996) *	0.973 (0.857–1.100)	0.904 (0.825–0.989) *	0.474 (0.437–0.514) *	0.838 (0.789–0.888) *
Practical work	/	1.044 (0.890–1.222)	1.406 (1.265–1.562) *	0.603 (0.547–0.663) *	1.058 (0.978–1.144)
**When the gesture is observed (reference: upon entering)**					
Upon exiting	0.918 (0.875–0.963) *	/	0.467 (0.435–0.501) *	/	/

* *p*-value < 0.05.

## Data Availability

The data that support the findings of this study are available from the corresponding author upon reasonable request.

## Appendix A

**Table A1:** Color codes in universities depending on the risk level associated with SARS-CoV-2.

Risk Level	Green (No Risk)	Yellow (Low Risk)	Orange (Moderate Risk)	Red (High Risk)
Interpretation of risk level	Vaccine available and/or herd immunity. Contact may occur. Hand hygiene is still necessary.	Limited spread of the virus. Contact is limited, but may occur depending on security conditions.	Systematic transmission of the virus. Contacts are limited to the essentials and take place when risk factors are under control.	Systematic transmission of the virus, contact is to be avoided as much as possible.
Occupancy of premises	Premises open and all services operational.	Premises open. Limitation to 75% of the maximum number of students possible. Services ensured by respecting all hygienic measures.	Premises open. Limitation to 20% of the maximum number of students possible. Services ensured by respecting all hygienic measures.	Premises open with minimal services provided.
Teaching and evaluation activities	Face-to-face activities possible.	Face-to-face and distance-learning.	Distance learning to be organized whenever possible.	Distance learning only.
Group size ≤ 50	No restriction.	Physical distancing of 1 m. Mandatory mask wearing.		Forbidden.
Groups of 51–200	No restriction.	Face covering and physical distancing of 1 m or occupation of 1 every 2 seats. Professor without mask if a physical distance of 3 m is maintained.	Face covering. Occupation of 1 every 5 seats. Professor without mask if a physical distance of 3 m is maintained.	Forbidden.
Groups > 200	No restriction.	Face covering and physical distancing of 1 m or occupation of 1 every 2 seats. Professor without mask if a physical distance of 3 m is maintained.	Forbidden.	Forbidden.
Movements	Free.	Unique traffic flow designated with arrows. Mandatory mask wearing.		
Restaurants	Free.	Opened with physical distancing of 1.50 m. and outside settings to be prioritized. Mandatory mask before and after eating.		Not accessible.

**Table A2:** Compliance rate for barrier gestures implementation and confidence interval from the first survey, 2020. Reprinted with permission from Ref. [20]. Copyright 2021, Renault et al.

Gesture	Total Number of Observations	Number of Compliant Observations	Compliance Rate (%)	Standard Error	Binomial Exact 95% CI
Greetings without contact	2768	2300	83	0.007	0.82–0.84
Hydro-alcoholic gel	8822	3868	44	0.005	0.43–0.45
Circulation flow	7335	4773	65	0.006	0.64–0.66
Mask wearing	10,856	8567	79	0.004	0.78–0.80
Physical distancing in of auditoriums	7266	6452	89	0.004	0.88–0.90
Physical distancing out of auditorium	3587	1585	44	0.008	0.43–0.46
